# Broadly Neutralizing Antibodies as Treatment: Effects on Virus and Immune System

**DOI:** 10.1007/s11904-017-0352-1

**Published:** 2017-03-27

**Authors:** Jinal N. Bhiman, Rebecca M. Lynch

**Affiliations:** 1 Centre for HIV and STIs, National Institute for Communicable Diseases (NICD), National Health Laboratory Service (NHLS), 1 Modderfontein Road, Sandringham, Johannesburg, Gauteng 2131 South Africa; 2 Faculty of Health Sciences, University of the Witwatersrand, Johannesburg, South Africa; 3 Department of Microbiology, Immunology and Tropical Medicine, George Washington University, 2300 Eye St. NW, Washington, DC 20001 USA

**Keywords:** Broadly neutralizing antibodies, Passive therapy, Virus escape, HIV-1, monoclonal antibodies

## Abstract

**Purpose of Review:**

The purpose of this study is to summarize recent advances in the use of broadly neutralizing antibodies (bNAbs) as therapeutics in human clinical trials and in non-human primate (NHP) models. We seek to highlight lessons from these studies with an emphasis on consequences to the virus and immune system.

**Recent Findings:**

In the past 10 years, advances in HIV-1 trimer structure and B cell isolation methods have precipitated the identification of “new-generation” anti-HIV antibodies with broad and potent neutralization. In the past 2 years, the concept of using these bNAbs as therapeutic tools has moved from NHP models into human clinical trials. These trials have investigated the effects of bNAb infusions into patients chronically infected with HIV-1, while the NHP model has investigated treatment during acute infection.

**Summary:**

Through this work, the relationship between in vitro breadth and potency and in vivo clinical effect, although unresolved, is gradually being elucidated. These results emphasize the need for combination antibody therapy.

## Introduction

Antibodies have been used as passive therapy for infectious diseases since the treatment of diphtheria with serum in the late 1800s [1]. Currently, two monoclonal antibodies (mAbs) are licensed for use against anthrax toxin and respiratory syncytial virus (RSV) F protein [1], and many other antibodies, including those against HIV-1, are currently in development as therapeutics. Of particular interest to the HIV-1 field, infusion of non-human primates (NHPs) with an α4β7 integrin targeting mAb was recently shown to be efficacious in suppressing simian-immunodeficiency virus (SIV) [2]. As for virus-specific antibodies, neutralization targets on the trimeric HIV-1 envelope (Env) are shielded from the immune response through extensive glycosylation and large variable loops found on these proteins [3]; therefore, a major goal of the HIV-1 vaccine field has been to identify areas of vulnerability on the trimer [4]. The isolation and characterization of broadly neutralizing antibodies (bNAbs) from HIV-infected individuals has highlighted a continuum of exposed sites on the trimer that will be useful for rational vaccine design [5] and has also garnered a variety of antibodies that could be valuable tools for HIV prevention and treatment [6, 7].

The feasibility of antibodies for treatment of HIV-1 depends on many factors including manufacturability, cost, and delivery, with current research investigating antibody development and optimization to exploit the full range of antibody-associated antiviral effects. Notably, all antibody functions (neutralizing and non-neutralizing) could likely contribute to adjunctive therapy with current anti-retroviral treatment (ART) regimens [4, 8]. Virus neutralization, however, is likely the most well-characterized antibody function during HIV-1 infection; therefore, neutralizing antibodies as treatment have thus far been most extensively tested. Previous clinical trials, which passively infused neutralizing mAbs into HIV-1-infected patients on or off ART, were conducted with antibodies 2G12, 2F5, 4E10, or KD-247, which are less broad and potent than “new-generation” mAbs. These antibodies were found to either decrease virus load (VL) or delay virus rebound only in a minority of subjects, and virus escape mutations were often detected [9–14]. Interestingly, in an analytic treatment interruption (ATI) study, it was found that antibody treatment during acute as opposed to chronic infection as well as sensitivity of virus to in vitro neutralization by mAbs appeared to delay virus rebound during ATI (i.e., increased effect of antibody) [13, 15].

**Fig. 1:**
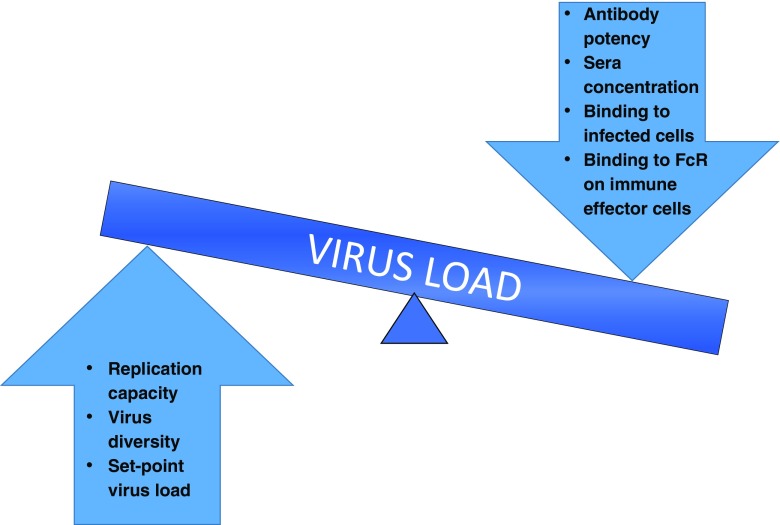
Potential effects on virus load after infusion with a monoclonal antibody. On the right are antibody-specific factors that can affect virus load while on the left are virus-specific factors

To date, the correlation between in vitro neutralization and in vivo clinical effect is not fully defined, and the effect of antibody treatment on the balance between the virus and immune system, with particular reference to viral escape and the effect on viral set point, remains unknown (Fig. 1). Viral escape from antibodies, which has been well documented during natural infection [4, 16, 17] even in individuals who develop bNAbs, occurs rapidly and via multiple routes [18–20]. In the context of bNAbs as therapy, the ease of viral escape as well as the number of bNAbs or bNAb specificities required to contain virus replication by limiting viral escape or reducing viral fitness, remains to be determined. These questions will be informed through ongoing clinical trials using new generation bNAbs and recently published work describing the infusion of bNAbs into chronically HIV-1-infected individuals both on and off ART as well as in the context of ATI. Meanwhile, the effects of bNAb infusion during early infection are being explored in NHP models. In this review, we will focus on the relationship between in vitro potency and in vivo effect, virus escape from bNAb pressure and effects on both viral load and the immune system in recent human clinical trials and lessons from the NHP models.

## Passive Infusion of bNAbs During Chronic SHIV Infection

Two studies demonstrated the feasibility of passive immunization with bNAbs during chronic infection with simian-human immunodeficiency viruses (SHIVs) in rhesus macaques (RMs). Both tested bNAbs, singly and in combination, that targeted the HIV-1 envelope receptor CD4 binding site (CD4bs) (3BNC117 and b12) or the V3-glycan supersite (10-1074 and PGT121). Because 3BNC117, 10-1074, and PGT121 either have or will be used in human clinical trials, a comparison between human and NHP data will be possible.

The first study by Shingai et al. infected RMs with SHIV_AD8_ and after 12 weeks of infection, during virus set-point, they infused four RMs with 10 mg/kg intravenously of either 3BNC117 or 10-1074 [21]. These antibodies exhibited potent neutralization of the infecting SHIV_AD8_ virus, with IC_50_ values ≤0.2 μg/ml. Infused RMs suppressed virus within 4–14 days and maintained suppression for 2–10 days. Single-genome amplification (SGA) of rebound virus on days 23 and 28 post-infusion revealed resistance mutations to 10-1074 (loss of N332 glycan), but no obvious changes within the 3BNC117 epitope. Antibodies were next co-administered to five RMs infected for at least 3 years. In the three RMs whose baseline VL was 1 × 10^4^ copies/ml or lower, virus was suppressed by day 6 or 7 and remained undetectable until rebound between days 17 and 42. In the two RMs with virus >1 × 10^5^ copies/ml, VL decreased until day 6 or 20 and then began to increase. SGA on rebound viruses on days 28, 49, or 57 revealed no obvious resistance mutations within antibody epitopes.

Barouch et al. reported similar results in SHIV_SF162P3_-infected RMs [22]. RMs were infected for 9 months before a single infusion dose of 10 mg/kg either of PGT121 (a clonal relative of 10-1074) or 3BNC117. Interestingly, although PGT121 potently neutralized the infecting strain SHIV_SF162P3_ in vitro, this virus was more resistant to 3BNC117. This observation correlated with viral suppression within 7 days and maintenance of suppression for 42–56 days in the four macaques infused with PGT121. Remarkably, in one of these PGT121-infused animals, viral control was observed for more than 200 days in the absence of antibody. The four macaques infused with 3BNC117, however, experienced a smaller decline in VL, without full suppression, and returned to set-point by day 20 post-infusion, reflecting the insensitivity of the virus to this bNAb. Combination therapy worked well, as demonstrated by the infusion of 10 RMs with a cocktail of anti-HIV bNAbs (five with PGT121, 3BNC117, and b12 and five with PGT121 and 3BNC117) and >2 log decrease in VL. The two RMs with the highest baseline VL (around 5 × 10^5^ copies/ml) did not fully suppress virus replication and VL began to increase around day 15. The other eight RMs remained suppressed for multiple days and began to rebound between days 20 and 84 post-infusion. Together, in all RMs infused with PGT121, the time to virus rebound was correlated with baseline VL. Despite these differences in virus rebound SGA of virus from seven RMs at week 8 post-infusion revealed no obvious resistance mutations, although changes in and around the epitopes of these antibodies could be detected. In addition, the effect of the 3BNC117 in these cocktail antibody infusions was debated due to its reduced capacity to neutralize the circulating virus.

This study highlighted hints of indirect effects of antibody treatment on the immune system and the virus. A trend of increased neutralization potency by autologous antibodies against SHIV_SF162P3_ after infused mAb was cleared, as well as reduced activation of virus-specific T cell responses and a slightly lower median set-point VL (0.61 log lower) was observed in antibody-treated RMs.

Together, these NHP studies demonstrate that viral escape from infused antibody can occur, but also that it is possible for a single bNAb to suppress virus replication. In this case, virus rebounds to set-point as antibody levels wane, with little evidence of virus escape. Furthermore, low baseline VL, high viral neutralization sensitivity, and bNAb combination therapy were associated with longer suppression of virus replication. However, within this NHP model, the development of anti-drug responses led to faster clearance of human antibody concentrations in the RM serum. In addition, the chronic SHIV quasi-species in the NHP model may be less diverse than HIV-1 in humans; therefore, neutralization sensitivity of the circulating virus may be easier to define in RMs leading to a clearer relationship between in vitro and in vivo potency.

## Passive Infusion of bNAbs During Chronic HIV-1 Infection

The first passive immunization clinical trial using new-generation bNAbs in humans intravenously infused CD4bs antibody 3BNC117 into 17 viremic subjects (presumably subtype B infected) with a dose escalation between 1 and 30 mg/kg [23]. Overall, the level of VL decline post-infusion correlated with antibody dose. Within the highest dose group of 30 mg/kg, five of the eight participants were pre-screened before infusion to ensure their virus was sensitive to 3BNC117, but all eight participants had decrease in VL between 0.8 and 2.5 log_10_. Mean VL decrease was 1.48 log_10_ and median nadir was 7 days. The magnitude of the decline was found to be related to the baseline VL and pre-existing sensitivity of the virus to the antibody. Sensitivity was tested by bulk PHA stimulation of autologous PBMC, and resulting virus outgrowth in the supernatant was used in the TZM-bl assay. No participant maintained full suppression even in the high antibody dose group; however, by 56 days, three of the eight had not yet fully returned to baseline VL (within 0.5 log_10_). These observations might suggest that virus escape from 3BNC117 occurred but that the virus may not replicate as well.

To test for virus escape, autologous virus sensitivity to 3BNC117 was tested from pre- and post-infusion time points. Increased resistance to 3BNC117 was detected even in the low-dose group, although the degree to which the virus became more resistant was variable. SGA was performed on pre- and post-infusion plasma virus *env* genes for a subset of individuals and Envs were cloned to test for antibody resistance. Overall, cloned Envs from plasma virus became more resistant to 3BNC117 post-infusion, but the number of cloned Envs (three per time point) was low for comparison. Unsurprisingly, the neutralization data from cloned Envs did not exactly match the virus outgrowth neutralization data, highlighting the differences between these two methodologies. When sequences of plasma *envs* were compared, changes could be detected in some subjects around the 3BNC117 epitope, especially in loop D and loop V5, but there were no consistent resistance mutations detected.

A second phase 1 clinical trial tested infusion of CD4bs bNAb VRC01 into chronically HIV-1-infected individuals and many of the findings were similar to the Caskey study [24]. Importantly, 3BNC117 and VRC01 target an overlapping epitope on the receptor binding site of the gp120 virus protein and are highly genetically and structurally similar despite being isolated from different donors [25–27]. Even so, slight differences between these antibodies lead to potency differences for subtype B viruses (Table 1). In this study, eight subtype B-infected participants with detectable viremia were intravenously infused with 40 mg/kg of VRC01. This infusion reduced VL decline between 1.1 and 1.8 log_10_ in six of the eight participants, and mean nadir for all eight was 9 days. Plasma Envs, cloned from before and after infusion, revealed that the disparate effects on VL decline were related to pre-existing virus resistance to VRC01. The two participants with little to no decline in VL had relatively high autologous Env IC_80_values (geometric mean IC_80_ of 10 cloned Envs were 30 and 17 μg/ml), and the level of infused antibody was calculated to be less than 100-fold above the mean IC_80_. Additionally, level of virus suppression appeared to be related to baseline VL because the two participants with the lowest baseline VLs (<1000 copies/ml) maintained virus suppression until very low to undetectable antibody levels were reached. Sequencing and cloning rebound virus from these 2 participants revealed no evidence for increased resistance to or escape from VRC01 suggesting rebound occurred due to low antibody levels [29]. The other four participants, whose VL decreased until day 9, experienced virus rebound in the presence of detectable VRC01 concentrations, and by day 56, the VL of all eight had returned to baseline levels. SGA of plasma *envs* revealed changes in the virus quasi-species between pre- and post-infusion. Many of these sequence differences were detected within the VRC01 epitope, especially changes in loop V5 length; however, no consistent resistance mutations were found. Cloned Envs were tested for increased resistance to other bNAbs that may be used in future clinical trials and no difference was detected, apart from slight increase in resistance to the CD4bs antibody 3BNC117.

**Table 1:** Breadth and potency of antibodies that may be used in clinical trials on an 80 virus panel organized by subtype as generated by CATNAP http://hiv.lanl.gov/catnap [28]

		10-1074	10E8	3BNC117	PGDM 1400	PGT121	VRC01	VRC07-523-LS	VRC 26.25
Subtype AE(*n* = 9)	% Virus Neutralized (IC_80_ < 10 μg/ml)	0	100	89	89	0	89	89	67
	Geometric Mean(IC_80_ < 10 μg/ml)	n/a	1.75	0.326	0.021	n/a	1.09	0.269	0.055
Subtype AG(*n* = 6)	% Virus Neutralized(IC_80_ < 10 μg/ml)	83	100	83	67	50	67	83	67
	Geometric Mean(IC_80_ < 10 μg/ml)	0.536	1.37	0.251	0.035	0.511	0.677	0.169	0.087
Subtype A1(*n* = 13)	% Virus Neutralized(IC_80_ < 10 μg/ml)	38	77	85	85	46	85	85	62
	Geometric Mean(IC_80_ < 10 μg/ml)	0.143	4.60	0.085	0.034	0.140	0.266	0.073	0.091
Subtype B(*n* = 15)	%Virus Neutralized(IC_80_ < 10 μg/ml)	87	100	87	67	80	93	100	13
	Geometric Mean(IC_80_ < 10 μg/ml)	0.154	2.22	0.260	0.449	0.236	0.941	0.234	0.538
Subtype C(*n* = 37)	% Virus Neutralized(IC_80_ < 10 μg/ml)	62	89	70	70	59	86	95	65
	Geometric Mean(IC_80_ < 10 μg/ml)	0.226	1.65	0.402	0.030	0.163	1.00	0.184	0.010

*n*/*a* not applicable

Together, these two clinical trials indicate that, similar to the NHP model, baseline VL and sensitivity to antibody can affect the degree to which an infused antibody suppresses virus (i.e., VL decline and time to return to baseline). A more complicated picture was formed in the human trials, where selection pressure against the most sensitive virus species may have been sufficient to allow VL increase in the presence of the antibody. While these reports examined bNAb effects on the virus quasi-species, antibody infusion may have affected the immune response as well.

## Effects of Passive Infusion on Immune Responses

One reason for use of antibodies as treatment is their potential to exert multiple anti-viral effects. Theoretically, infusion with bNAbs could not only neutralize circulating plasma virions but also bind infected cells for Fc-mediated clearance as well as form immune complexes that could enhance antigen presentation. A follow-up study of the 3BNC117 clinical trial examined the possibility of enhanced immune function following bNAb infusion by analyzing sera and virus 6-month post-infusion, once 3BNC117 was no longer detectable in serum [30]. IgG was purified from sera pre- and 6-month post-3BNC117 infusion, and consistent with antibody evolution during natural infection [31–37], 6-month IgG could neutralize these pre- and 1-month post-infusion viruses better than pre-infusion IgG. These data confirm that the autologous B cell response remains evolving against infecting virus even in the presence of infused antibody, which may be attributed to extended presence of virus in the germinal centers despite VL decrease. Next, 6-month IgG was tested against a heterologous panel of globally circulating strains [38] and slightly increased neutralization was found compared to pre-infusion IgG. To note, these data were measured as area under the curve (AUC) instead of inhibitory concentration (IC_50_ or IC_80_) as is the standard in the field for reporting neutralization data. The use of AUC to quantify neutralization incorporates viruses that are not neutralized up to 50% into the dataset, thereby allowing measurement of subtle shifts in neutralization; however, it also allows measurement of noise within the neutralization assay. Examination of the reported IC_50_ values with a more stringent cutoff reveals significant differences (above the three-fold error of the neutralization assay in more than one virus) in one of 15 infused viremic individuals, none of the infused individuals on ART, and one of the 18 control sera (chronically infected individuals who were not infused with the bNAb). Within the control individuals, 13 possessed bNAb activity in the pre- and post-infusion samples, which may have confounded this observation. This finding of neutralization improvement in infused participants did not correlate with CD4 level, VL, or pre-infusion neutralization capacity. Longitudinal SGA of the virus quasi-species in a subset of individuals detected inconsistent changes in diversity post-infusion, and confirmed that, similar to previous findings [39–41], the level of pre-infusion virus diversity correlated with pre-infusion IgG neutralization capability. Because the measurement of virus diversity may not capture minor changes within the quasi-species, the 3BNC117 epitope was specifically observed longitudinally and again, changes in all subjects were detectable but none were uniform between subjects. Mapping to identify the targets of these improved responses and diversity within those specific epitopes may be more informative in future studies.

The mechanisms behind this phenomenon of augmented neutralization may include increased virus diversification in the presence of heterologous antibody leading to new B cell stimulation or the presence of increased immune complexes that serve as immunogens expanding B cell responses. Interestingly, the virus in subject 2C4 was resistant to 3BNC117 and VL did not decline in the presence of the antibody [23], but there was an augmentation of IgG neutralization, suggesting that a robust number of immune complexes are not necessary for this boost. Additionally, the two participants (2C4 and 2A1) with the greatest envelope diversity and breadth on the virus panel pre-infusion were the two with the greatest change in IC_50_, suggesting that breadth may have been naturally developing. Altogether, observations of modest increases in breadth and potency after infusion with a heterologous antibody, while intriguing, remain to be fully explored and confirmed.

## Effect of Baseline Virus Load on Virus Suppression During Passive Infusion

Recent studies of ATI allow assessment of the ability of antibodies to suppress virus when baseline virus is undetectable (i.e., fully suppressed on ART). A paper published by Scheid et al. describes the results of a phase IIa open label clinical trial where 13 participants, with VL <50 copies/ml for over 12 months, were infused multiple times with antibody 3BNC117 and discontinued ART 2 days after the first infusion [42]. Before infusion, participants were screened by virus outgrowth culture and TZM-bl assay to measure pre-existing sensitivity to 3BNC117, and only those whose outgrowth viruses had IC_50_ ≤2.0 μg/ml to 3BNC117 were enrolled. The average time to rebound was 8.4 weeks, and this result was significantly different from the 2.6 weeks in matched historical controls, from previous AIDS Clinical Trials Group (ACTG) ATI studies. In this study, time to rebound did not correlate with pre-ATI virus outgrowth sensitivity to 3BNC117, with cell-associated HIV-1 DNA, or with sera concentrations of infused antibody. It is important to note that because all participants were pre-screened for sensitivity to 3BNC117, correlation between pre-infusion sensitivity and time to rebound may not be possible to measure in this analysis. Rather, it is conceivable that once the virus has reached some threshold of sensitivity, differences in time to rebound may be difficult to detect. Interestingly, participant 711 had the shortest time to rebound despite 3BNC117-sensitive virus as measured by IC_50_ or IC_80_, but this pre-infusion virus was incompletely neutralized (with a maximum percent inhibition of 95%) at the highest concentration of antibody (50 μg/ml). This observation highlights the importance of in vitro pre-infusion virus sensitivity to clinical outcome during passive therapy, with particular emphasis on incomplete neutralization, a common trait among all bNAb classes [43].

Four subjects, whose virus did not become more resistant to 3BNC117, rebounded in the presence of low antibody concentration. The authors calculated that VL became detectable in these individuals only when antibody concentrations waned to less than 10-fold above the mean IC_80_ of the virus. This observation supports the notion that for certain individuals and perhaps particular viral genetic determinants, monotherapy can suppress virus replication if a certain level of antibody is maintained. SGA and cloning of Envs from plasma during virus rebound in seven participants confirmed that, for the majority of subjects, rebound virus was more resistant to 3BNC117 compared to pre-infusion virus outgrowth culture. It should be noted, however, that comparisons between pre- and post-infusion virus are difficult in low-plasma virus settings. The authors phylogenetically compared the sequences of a few virus outgrowth cultures to rebound plasma virus sequences, because pre-ART plasma samples were unavailable. Despite this limited sampling, it is clear that post-ATI plasma rebound virus was overall monophyletic in the eight participants analyzed. Again, analysis of rebound virus found residue changes within the epitope of 3BNC117 in loop V5 or loop D that could be associated with resistance; however, there was no consistent pattern of change. Rebound viruses were tested for increased resistance to other antibodies that may be used in clinical trials and generally no difference was found.

Two subsequent trials (NIH 15-I-0140 and ACTG A5340) tested the ability of VRC01 infusion to delay virus rebound during ATI; however, in these trials, which examined 10 and 13 participants respectively, pre-screening for VRC01-sensitive virus was not performed [44]. The median time to rebound in these studies was 4 weeks (A5340) and 5.6 weeks (NIH). This delay was calculated to be significantly different from historical ACTG controls at 4 weeks post-ATI but by 8 weeks, there was no difference. SGA-derived plasma virus *env* genes from eight participants in the ACTG trial were cloned from both before ART and after ATI time points. Strikingly, in some participants, there was a clear monophyletic rebound virus suggesting genetic restriction in the presence of VRC01, and rebound virus was demonstrated to be more resistant to the antibody in six of these individuals. Interestingly, the two subjects with the longest time to rebound in this study were the two with the most VRC01-sensitve virus before ART, and in most other subjects, pre-existing resistance in virus populations could be detected. The NIH trial measured the neutralization sensitivity of replication-competent viral isolates from autologous CD4 T cells before and after VRC01 infusion, and indeed, this study found increased resistance to VRC01 in six of seven subjects tested, which was significantly different in four.

Together, these studies highlight the impact of pre-screening virus for in vitro sensitivity as an indicator of greater in vivo clinical effect and certainly suggest that for monotherapy with antibodies, pre-screening may be of greater consequence than for ARV drug resistance. Furthermore, there is evidence for genetic restriction of rebound virus during ATI in the presence of antibody, which although not clinically relevant does indicate viral selection pressure by the infused antibody. Finally, the lack of resistance signatures discovered in these studies may reflect the complex nature of the CD4bs epitope, which may be true for CD4bs antibodies in general.

## Optimization of Passive Infusion with bNAbs: Timing, Delivery, and Product

The question of timing of antibody treatment is now being investigated in the SHIV-NHP model where combination antibody treatment during early infection (Fiebig I–II) was demonstrated to decrease VL at the same pace as cART treatment [45]. This study by Bolton et al. also hinted at increased immune function in the presence of antibody-immune complexes, an observation that has not yet been fully developed. An exciting recent study by Hessell et al. demonstrated that combination antibody treatment given to infant RMs 24 h post-exposure could prevent systemic infection [46], the first demonstration of virus clearance by bNAbs. These studies of early bNAb treatment may be difficult to replicate in humans but similar strategies are currently being tested in clinical trials such as IMPAACT 2008 and RV398.

Optimizing methods of antibody delivery are currently being explored, including vectored antibody genes. Adeno-associated virus (AAV) delivery of bNAbs as well as CD4 mimetics has demonstrated protection in animal models, but may not be feasible as a therapeutic strategy in the context of antiretrovirals [47, 48]. Increasing potency and breadth by engineering bi-specific antibodies may be a successful strategy to both improve function and manufacturability (one product with a combination of antigen-binding domains). One study that combined two different bNAb Fabs into a single bi-specific antibody established that this single product recapitulated breadth and potency of the two parental bNAbs combined [49]. Further reports have demonstrated that strategies to combine bNAb 10E8 with anti-CD4 mAb ibalizumab or to combine multiple bNAb Fabs such as 3BNC117 and PGT135 in an IgG3 constant region construct can successfully increase neutralization breadth and potency as a single mAb [50, 51]. Thus, many variations of bNAbs will be able to be tested for therapeutic effects in the near future.

## The Whole Antibody: Fc and Fab

While this review is focused on the neutralization breadth and potency of bNAbs, which is mainly derived from their antigen-binding variable region (Fv), the whole antibody, variable and constant region (Fc) together, can exert anti-viral effects beyond neutralization. Antibodies can tag virions or infected cells for phagocytosis or killing, and these processes involve Fc binding to Fc receptors (FcR) on phagocytic or effector cells. In a setting of chronic HIV infection, passive infusion of bNAbs may greatly facilitate these effector functions that an otherwise exhausted and dysfunctional B cell response may not be capable of. Passive mAb therapy provides an opportunity to engineer and optimize half-life or polyfunctionality through Fc mutations, glycosylation patterns, and the use of particular isotypes as reviewed in [28, 52]. In particular, antibody-dependent cellular cytotoxicity (ADCC) requires not only antibody Fc binding to effector cells but also variable region binding to infected cells. Importantly, however, epitope availability and conformation may be very different on infected cells than on virions, and the ability of a bNAb to neutralize virus does not necessarily confer the ability to recognize and bind infected cells for killing. A recent study by Bruel et al. compared bNAbs expressing the same Fc region for their ability to mediate ADCC of a cell line infected in vitro with either of two lab-adapted strains of HIV-1 [53]. They reported that neutralization potency, binding to infected cells, and stability of that binding all correlated with the ability of the bNAb to mediate ADCC. Thus, future directions for mAbs as therapeutics include optimizing all regions of the antibodies for functions beyond neutralization.

A role for bNAbs has also been implicated in HIV-1 cure strategies, which have focused on methods to reverse latent virus (“shock”) and to mobilize the anti-HIV-1 immune response (“kill”). Taking antibody engineering one step further, many groups have followed the path of cancer immunotherapy by designing two-armed molecules that combine one HIV-recognizing arm with an arm that can activate effector cells These molecules mainly utilize two formats: bi-specific T cell engagers (BiTEs) and dual-affinity re-targeting (DART) molecules. Both formats have been tested using Fabs of bNAbs as the HIV-specific arm; however, the measurement for these assays is killing of infected cells, not neutralization. One study by Pegu et al. constructed BiTEs using the Fab region of bNAb VRC07-G54W linked to an anti-human CD3 single-chain variable fragment and found that the BiTE could activate and lyse latently infected cell lines [54]. Another study constructed DARTs containing an anti-human CD3 arm and an anti-HIV Fab, but tested Fabs of four bNAbs and two non-NAbs for their ability to induce CD8 killing of autologous in vitro-infected CD4 T cells [55]. Interestingly, the authors found that VRC01 and 10E8 DARTs could not mediate very potent killing whereas the DART containing the PGT121 Fab was very effective. Furthermore, this DART, in combination with PKC agonist indolactam, was shown to greatly reduce the inducible reservoir in the cells of at least one of eight cART-treated HIV-1-infected individuals tested. This study highlights the fact that not all bNAbs are able to effectively bind HIV-1-infected cells, but testing different combinations of Fabs in different molecular formats will surely be further examined.

## Conclusions and Future Directions

Overall, data in human clinical trials support the observations from NHP models that infusion of heterologous bNAbs during chronic infection can exert significant effects on the virus; however, factors such as potency of neutralization and baseline VL affect the outcome. Pre-screening participants for antibody sensitivity presumably led to increased observed clinical effects (better VL decline in viremic subjects and longer time to rebound during ATI) [42]. While the increased efficacy of 3BNC117 as compared to VRC01 could be attributed to potency or half-life, the reported half-lives of 3BNC117 and VRC01 are not very different [23, 24]. The role of half-life in antibody efficacy may be tested in the future through the addition of the half-life increasing “LS” mutations in the antibody Fc region [56]. With respect to potency, the relationship between clinical effect and pre-existing virus sensitivity highlights the need to consider effective antibody concentrations as relative (i.e., a certain level above in vitro IC_80_) rather than absolute (need to be >100 μg/ml), with particular importance on prevalent subtypes in study populations. The clinical trials described here were performed in North America with predominantly subtype B-infected individuals. Data to address differences in subtype sensitivity may result from the current HVTN Antibody Mediated Protection Trials, which will infuse VRC01 into US and African populations which will infuse VRC01 into HIV negative US and African populations for prevention as opposed to HIV therapy.

The human trials described here have invaluably demonstrated that virus selection occurs in the presence of heterologous antibody and emphasize the need for combination antibody therapy in the future. It will be important to address optimizing bNAb combinations for geographic subtype [57] and analyzing pre-existing resistance to all bNAbs included in the cocktail. Continued investigation into the potential use of antibodies, as immunotherapeutics with fewer adverse effects, is warranted as antibodies offer alternate choices for the treatment of HIV-1-infected children or individuals with severe reactions to ART. NHP and human trials certainly highlight the potential effectiveness of bNAbs as therapy for HIV-1 infection and further investigation into the caveats associated with viral sensitivity and escape should be addressed in current and future clinical trials.
